# Dual-color deep-tissue three-photon microscopy with a multiband infrared laser

**DOI:** 10.1038/s41377-018-0012-2

**Published:** 2018-06-06

**Authors:** Khmaies Guesmi, Lamiae Abdeladim, Samuel Tozer, Pierre Mahou, Takuma Kumamoto, Karolis Jurkus, Philippe Rigaud, Karine Loulier, Nicolas Dray, Patrick Georges, Marc Hanna, Jean Livet, Willy Supatto, Emmanuel Beaurepaire, Frédéric Druon

**Affiliations:** 1 Laboratory Charles Fabry, Institut d’Optique Graduate School, CNRS, Université Paris-Saclay, 91128 Palaiseau, France; 2 Laboratory for Optics and Biosciences, Ecole Polytechnique, CNRS, INSERM, 91128 Palaiseau, France; 3 Vision Institute, CNRS Sorbonne Universités, Université Paris 6, INSERM, 75012 Paris, France; 4 Zebrafish Neurogenetics Unit, Developmental and Stem Cell Biology Department, Institut Pasteur, CNRS, 75015 Paris, France

## Abstract

Multiphoton microscopy combined with genetically encoded fluorescent indicators is a central tool in biology. Three-photon (3P) microscopy with excitation in the short-wavelength infrared (SWIR) water transparency bands at 1.3 and 1.7 µm opens up new opportunities for deep-tissue imaging. However, novel strategies are needed to enable in-depth multicolor fluorescence imaging and fully develop such an imaging approach. Here, we report on a novel multiband SWIR source that simultaneously emits ultrashort pulses at 1.3 and 1.7 µm that has characteristics optimized for 3P microscopy: sub-70 fs duration, 1.25 MHz repetition rate, and µJ-range pulse energy. In turn, we achieve simultaneous 3P excitation of green fluorescent protein (GFP) and red fluorescent proteins (mRFP, mCherry, tdTomato) along with third-harmonic generation. We demonstrate in-depth dual-color 3P imaging in a fixed mouse brain, chick embryo spinal cord, and live adult zebrafish brain, with an improved signal-to-background ratio compared to multicolor two-photon imaging. This development opens the way towards multiparametric imaging deep within scattering tissues.

## Microscopy: Looking deeper with three photons

Researchers in France are using a novel infra-red light source to examine both fixed and living tissue samples in deeper detail than previously possible with a technique called three-photon microscopy. The absorption of three photons at different infra-red frequencies stimulates subsequent emission of light from fluorescent molecules in the sample. Detecting the fluorescence by microscopy reveals the location and interactions of the molecules concerned. Emmanuel Beaurepaire and Frederic Duon at the University of Paris-Saclay, developed a procedure to emit ultra-short pulses of infra-red laser light with optimal characteristics for three-photon microscopy. They demonstrated their innovation by studying fluorescent proteins in brain and nerve tissue taken from mice and chicks, and also in live zebrafish brain. The procedure offers opportunities to study molecular structures and interactions more effectively than previously possible with three-photon microscopy.

## Introduction

Multiphoton microscopy^1^ is now established as the reference method for both deep and live fluorescence imaging of biological tissues. Indeed, such an approach delivers a sub-cellular resolution at depths of hundreds of micrometers inside intact tissues. Together with the rapid progress in genetically engineered probes, two-photon microscopy is a key enabling technology in fields such as neuroscience, developmental biology, immunology, and others. However, tissue penetration for two-photon microscopy is limited by scattering^2,3^. When laser power is not a limiting parameter, compensating for the exponential decrease in unscattered light with depth results in out-of-focus fluorescence, which degrades the signal-to-background ratio and effectively limits the imaging depth^3,4^. One recently demonstrated effective strategy for deeper multiphoton imaging is to use three-photon (3P) excitation while shifting the excitation to the short-wavelength infrared (SWIR) range to approximately 1300 nm (ref. ^5^) or 1700 nm (ref. ^4^). This strategy has two key advantages: (i) when the laser is focused at a depth equivalent to several times the scattering mean free path inside the tissue, 3P excitation shows a greatly improved rejection of the out-of-focus fluorescence background^3,4^; (ii) the wavelength windows at approximately 1300 and 1700 nm offer a better combination of tissue scattering and absorption properties compared to the 700–1100 nm wavelength range commonly used in two-photon-excited fluorescence (2PEF) microscopy^4^, enabling superior penetration. In addition, 1300 nm was found to be a nearly optimal wavelength for 3P excitation of green fluorescent protein (GFP) and derived calcium indicators^5^, and 1700 nm is appropriate for 3P excitation of widely used genetically encoded red probes, such as red fluorescent protein (RFP) and tdTomato^4,6^. Due to the weakness of 3P absorption cross-sections, however^7,8^, the pulsed excitation regime typically used for 2P microscopy (80 MHz, 100 fs, up to 2 nJ pulses at the sample surface) is not appropriate for 3P microscopy. Instead, pulse trains in the MHz, sub-100 fs, and few hundreds nJ range are necessary to realize rapid deep-tissue 3P imaging while minimizing tissue heating^9,10^.

**Fig. 1:**
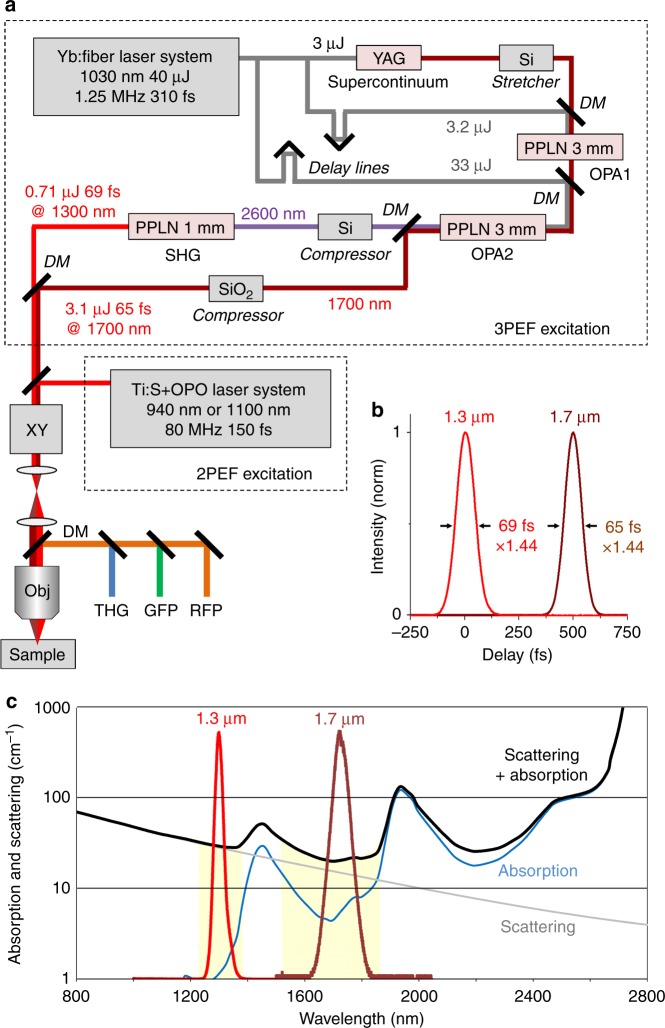
Dual-band SWIR laser source optimized for three-photon microscopy. **a** Experimental setup showing the source design. A Yb:fiber laser providing 1030 nm pulses at 1.25 MHz is used for supercontinuum generation in a YAG crystal and for amplification in a two-stage OPCPA arrangement. Signal and idler beams are produced at 1.7 and 2.6 µm and the idler is frequency-doubled, resulting in simultaneous emission at 1.7 and 1.3 µm with pulse energies in the µJ range. The beams are injected into a scanning microscope for three-photon microscopy. Alternatively, an 80 MHz pulse train at 920 or 1100 nm is used for comparison with two-photon excitation. DM dichroic mirrors, OPA optical parametric amplification stages, *XY* beam scanning, Obj microscope objective. **b** Measured temporal profiles for the two SWIR beams. **c** Parameters limiting deep-tissue imaging in multiphoton microscopy. The solid black curve combining tissue scattering and water absorption indicates the interest in use of the 1.3 and 1.7 µm wavelength ranges for in-depth imaging. The red and brown graphs reproduce the measured spectra for our source outputs, targeting the spectral regions of interest

Optimized laser sources are required to develop such an imaging approach to its full potential^6,11–13^. In particular, as many potential applications require study of interactions between cells/tissue components labeled with different fluorophores, one important challenge is to develop laser sources that enable efficient two-color three-photon imaging. Given the wavelength separation between the SWIR bands relevant for 3P microscopy (1.3 and 1.7 µm), a single excitation band is insufficient for simultaneous imaging of, for example, GFP-labeled and RFP-labeled structures.

In this article, we report on the first multiband SWIR source that is able to simultaneously emit ultrashort pulses at 1.3 and 1.7 µm with characteristics appropriate for 3P microscopy (sub-70 fs duration, 1.25 MHz repetition rate, and µJ-range pulse energy). This design, which is based on optical parametric chirped-pulse amplification (OPCPA), outperforms current alternative sources and has intrinsically stable characteristics. The combined output enables simultaneous 3P fluorescence imaging of various combinations of GFPs and RFPs along with label-free third-harmonic generation (THG) imaging^14–16^ of the tissue structure. We demonstrate in-depth dual-color three-photon imaging in fixed mouse brains and in live developing spinal cord explants and compare these data with those obtained by two-photon microscopy.

## Materials and methods

### OPCPA implementation

For the simultaneous generation of 1.3 and 1.7 µm pulses, we started with a pump beam at a wavelength of 1.03 µm provided by an Ytterbium-doped fiber amplifier (YDFA) (Satsuma HP3, Amplitude Systemes) and implemented a two-stage OPCPA. We generated a signal beam at 1.7 µm and therefore a simultaneous idler beam at ≈2.6 µm, which we then frequency-doubled to obtain 1.3 pulses. The input signal to the OPCPA was obtained by supercontinuum (SC) generation driven by the pump pulses at 1.03 µm, resulting in a spectrum extending up to 2 µm (Supplementary Figure S1). We optimized SC generation by moving the YAG crystal along the focal plane and measuring the output power after a 1400-nm-long pass filter. We estimated the seed energy contained in the 1.6–1.8 spectral band to be 1 nJ.

The optical parametric amplification (OPA) stages were composed of two MgO:PPLN crystals (30.05 µm poling period) mounted into ovens operating at 115 °C. The first crystal was 3 mm long with a 10 × 1 mm^2^section, optimized to obtain a gain of 130 for a pump peak irradiance of 50 GW/cm^2^ and an energy of 3.2 µJ. The second crystal was a 3-mm-long MgO:PPLN with a 12 × 3 mm^2^ section and an aperture scaled up to sustain 33 µJ pump pulses while avoiding the onset of beam distortions. The pump and signal beam diameters at 1/*e*^2^ in the first OPCPA crystal were 190 × 180 and 270 × 260 µm^2^, respectively. The evolution of signal pulse energy with respect to pump energy is shown in Supplementary Figure S1. We observed a ring-shaped distortion of the output beam spatial profile at pump energies exceeding 3.2 µJ, corresponding to pump powers >4 W. The threshold for the appearance of this degradation depends on the average and peak power and may be attributed to thermal effects^17^ and nonlinear absorption as reported in ref.^18^. Alternatively, linear absorption, the photorefractive effect and pyroelectric effects could account for this beam distortion at high pump power^19^. The optimum pump energy eventually used in the first stage was 3.2 µJ, corresponding to a pump irradiance of 50 GW/cm^2^ and resulting in a gain of 130.

In the second nonlinear crystal, beam sizes were scaled up to accommodate the available pump energy of 33 µJ. The pump and input signal beams were collimated with diameters of 2 × 1.7 and 1.8 × 1.6 mm^2^, respectively, enabling operation at full energy without the onset of ring-like beam distortions.

To reach the wavelength of interest of 1.3 µm, second-harmonic generation (SHG) for the idler pulses was carried out in a third MgO:PPLN crystal. The idler was focused by a 100-mm CaF_2_ lens in a crystal with a poling period of 35.8 µm and a working temperature of 30 °C. Since the 2.6 µm idler beam can be perturbed by air humidity, we minimized the propagation distance between the OPA and frequency-doubling stage. The overall efficiency of the SHG process was 32%. This efficiency was limited by the parasitic SHG of the 1.3 µm beam because the poling period was close to the third-order quasi-phase matching for this interaction. The average power generated at 650 nm under standard operating conditions was 500 mW compared with 890 mW at 1.3 µm. Finally, the 1.3 and 1.7 µm beams were recombined using a dichroic mirror (HR1300/HT1700, Altechna, Vilnius, Lithuania).

### Multimodal multiphoton imaging

Imaging was carried out using a lab-built laser scanning microscope. The excitation source was either the dual-band OCPCA described in this article or a Ti:Sapphire + optical parametric oscillator (OPO) chain optimized for two-photon microscopy (Chameleon Ultra2 and MPX, Coherent, CA, USA). Source selection was achieved using a movable mirror. Power in the four beams was independently controlled using motorized wave plates and polarizers. The beams sizes at the microscope input were controlled with adjustable two-lens telescopes. Scanning was carried out using galvanometric scanners (series 6215, Cambridge Technology, Bedford, MA, USA). Excitation beams were focused into the sample using a water immersion objective optimized for IR transmission (×25, 1.05NA, XLPLN25XWMP2, Olympus, Tokyo, Japan). The objective transmission was higher than 65% at all of the wavelengths used. Scanning and acquisition were synchronized using lab-written LabVIEW software and a multichannel I/O board (PCI-6115, National Instruments, Austin, TX, USA). Signals were detected in the backward (epi) direction by GaAsP modules (H7422P-40, Hamamatsu, Japan) using lab-designed electronics enabling mixed counting/analog detection. Fluorescence and harmonic light was collected using a dichroic mirror (BLP01-830R, Semrock, Rochester, NY, USA) and directed toward three independent detectors using dichroic mirrors (Semrock 495 and 561 nm). Bandpass filters were used in front of the detectors to collect blue (Semrock 433/24), green (Semrock 520/15), and red (Semrock 607/70) light. Excitation power was increased with the imaging depth (see Supplementary movies, legends). We note that the dispersion introduced by the microscope components was not fully compensated during the imaging experiments. We optimized the duration of the 1700 nm pulse at the focus of the objective by incorporating silicon slabs of various thickness into the beam path while monitoring the THG signal obtained from a glass–water interface. We measured the pulse duration of the 1300 nm pulse at the focus using a microscopy autocorrelator (CARPE, APE, Germany). Overall, we estimated that the pulse durations at the focus were approximately 80–100 and 130–150 fs for the 1700 and 1300 nm pulses, respectively. The pixel dwell time was typically 5 µs, i.e., the acquisition times were typically 1 to 4 s for a 500 × 500 pixel image.

### Image analysis and processing

Data were analyzed using FIJI. Flat-field correction was applied to the three-channel (red/green/THG) time-lapse dataset to correct for illumination inhomogeneity. For each channel, the illumination profile was computed by averaging all of the intensity images for the t-stack followed by Gaussian blurring. Each image was then divided by the corresponding normalized illumination profile.

### Cell experiments

HEK 293 cells were grown for 24 h in 6-well plates (5 × 10^5^cells per well) and transfected with 1 µg of DNA plasmids encoding either EGFP, mCherry, or tdTomato under the strong CAG or *CMV* promoter using Lipofectamine 2000 (Invitrogen). After 24 h, transfected cells expressing GFP and one of the red FPs were mixed at a 1:1 ratio, deposited onto a 13 mm glass coverslip coated with collagen (50 µg/mL, Sigma), and cultured for an additional 24 h. The cells were fixed with 4% paraformaldehyde (PFA, Antigenfix, Diapath) and mounted in Vectashield medium (Vector laboratories).

**Fig. 2:**
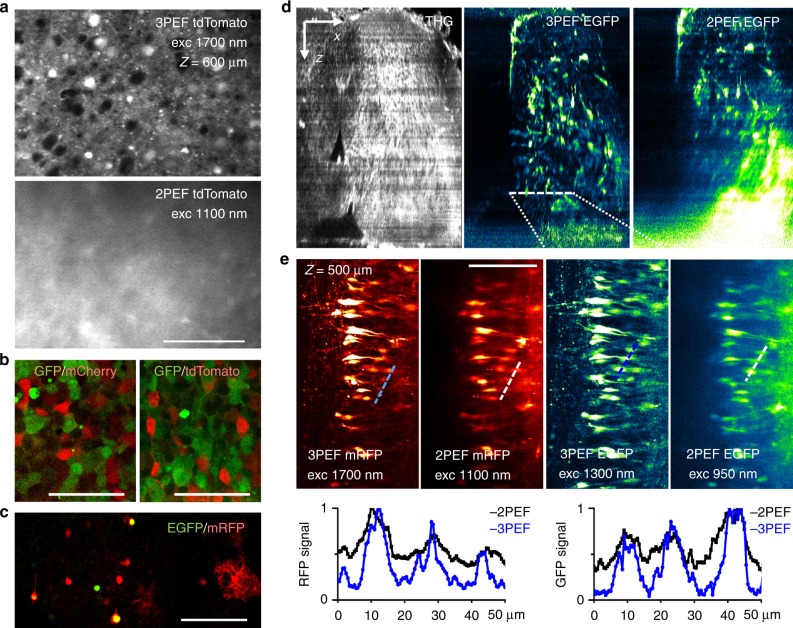
Dual-color and in-depth 3P imaging of nervous tissues. **a** Comparison of 3P and 2P excitation for imaging mouse brain tissue. 3PEF and 2PEF imaging of a tdTomato-labeled fixed mouse brain cortex at depths of 200 and 600 µm. See also Movie 1. **b**, **c** Dual-color 3PEF imaging for several combinations of green-red fluorescent proteins in **b** HEK cells and **c** mouse brain tissue at a depth of 500 µm. **d**, **f** Correlative 2PEF, 3PEF, and THG imaging of an intact chick embryo spinal cord (stage E9) co-labeled with EGFP and mRFP. Fluorescence images in each *XY* plane were normalized after acquisition for contrast comparison. See also Movies 2–4, and related information. **d** *XZ* projections of the THG, 3PEF EGFP, and 2PEF EGFP image stacks show the general morphology of the sample and the loss of 2PEF contrast with depth. **e** 3P and 2P mRFP and EGFP images recorded at a depth of 500 µm. Intensity profiles measured along the dashed lines illustrate the superior contrast provided in both channels by 3PEF excitation. Scale bars and arrows, 100 µm

### Mouse brain samples

Animal procedures were carried out according to the institutional guidelines. Mice were housed in a 14 h light/10 h dark cycle with free access to food.

To compare the performance of 3PEF vs. 2PEF for RFP imaging, we used a transgenic mouse line expressing a *Cytbow* transgene^20^ under the broadly active CAG promoter. Cre recombination, induced by intercrossing with *Emx1*^*IRES-Cre*^ mice^21^, yielded expression of tdTomato throughout the cerebral cortex of the resulting offspring. An 1-month-old animal was deeply anesthetized with sodium pentobarbital (150 mg/kg) and perfused transcardially with 4% PFA. After dissection, the brain was postfixed in PFA and mounted in 3% agarose. The block of tissue was immersed in phosphate-buffered saline (PBS), and the agarose covering the cortex was sliced off prior to imaging of the cortical surface.

To generate brain samples co-expressing RFPs and GFPs, we used in utero electroporation as previously described^20^. Briefly, timed-pregnant females (E13) were anesthetized with ketamine/xylazine and a midline laparotomy was carried out to expose uterine horns. One microliter of DNA transfection mix was injected with a glass capillary pipette into the lateral ventricle of the embryos. To maximize the number of labeled cells, we used DNA transposable elements based on the Tol2 and PiggyBac transposition systems, respectively, encoding a nuclear-addressed cytoplasmic GFP (*Tol2-CAG-H2B-GFP*, 1 µg) and a cytoplasmic RFP (*PiggyBac-CAG-mRFP*, 1 µg) along with plasmids encoding the corresponding transposases (*CAG-Tol2ase* and *CAG-PBase*, 0.2 µg). DNA was combined 9:1 with Fast Green dye (Sigma). An anode with a diameter of 3 mm, Tweezertrodes (Sonidel Limited), was placed above the dorsal telencephalon before the application of three 35 V pulses with a 50 ms duration, followed by three additional pulses in the reverse orientation. The incision site was then closed with sutures (4-0, Ethicon) and mice were allowed to recover in a clean cage. A brain from a P15 pup was prepared and mounted in agarose as described above. The block was sectioned into two parts in the coronal orientation at mid-brain level, and imaging of the cerebral cortex carried out on the resulting surface.

### Chick embryo spinal cord samples

Electroporation in the chick neural tube was performed at embryonic day 2 following the procedure described in a previous study^22^. Briefly, DNA was injected with a glass capillary in the neural tube and five 50 ms pulses of 25 V with 100 ms interval were applied using a pair of 5 mm gold-plated electrodes (BTX Genetrode model 512) separated by 4 mm and a square-wave electroporator (Nepa Gene, CUY21SC).

For fixed spinal cord samples, plasmids encoding EGFP (*CAG-EGFP*) and mRFP (*CAG-mRFP*) were co-electroporated at an equal concentration (1 µg/µL). After fixation in 4% PFA, E9 spinal cords were dissected and pinned on a silicone-coated dish filled with PBS, with the dorsal horn on top, which was positioned under the microscope objective for imaging.

For live imaging experiments, a plasmid encoding the proneural gene Neurog2 (*CAG-Neurog2*, 1 µg/µL) was used to induce neural differentiation; this plasmid was co-transfected with *CAG-EGFP* (1 µg/µL) and *CAG-H2B-mRFP* (0.5 µg/µL) to visualize the cell cytoplasm and chromatin, respectively. Embryos were harvested one day after electroporation, transferred to F12 medium, and slit along their midline from the hindbrain to the caudal end. The electroporated side of the neural tube was peeled off and mounted in a glass-bottom culture dish (MatTek, P35G-0-14-C) using a thin layer of 1% low-melting agarose dissolved in F12 medium. After polymerization of the agarose, the dishes were filled with 3 mL of culture medium (F12/penicillin streptomycin/sodium pyruvate) and transferred to a heating plate (37 °C) positioned under the microscope objective.

**Fig. 3:**
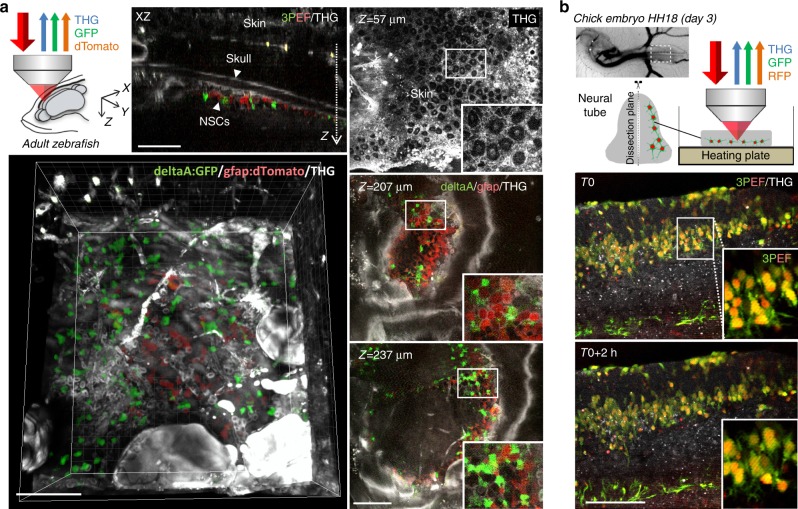
Live dual-color 3PEF and THG neural tissue imaging. **a** In vivo imaging through the skull in adult zebrafish telencephalon. The figures show representative *XY*, *XZ*, and 3D views for a volume encompassing two labeled cell populations: red dTomato-labeled radial glia and green GFP-labeled neural stem cells expressing the deltaA neurogenic gene (see Materials and methods section). Simultaneously acquired THG signals provide additional label-free contextual information (skin and skull morphology, blood vessels, lipid accumulations). See also Movies 7 and 8 and related information. **b** Simultaneous dual-color 3PEF and THG imaging of developing chick embryo spinal cord tissue (stage E3) expressing cytoplasmic GFP labeling and nuclear RFP labeling. The images are extracted from a 2-h-long experiment and illustrate developmental processes such as cell migration and process formation. See also Movies 5 and 6 and related information. Scale bars, 100 µm

**Telencephalon live imaging in adult zebrafish**

Three-month-old adult zebrafish (*Danio rerio*) were used. The transgenic line was created by crossing Tg(*gfap:dTomato*)^23^ with Tg(*deltaA:GFP*)^24^ in a casper double mutant background (roy−/−;nacre−/−)^25^ and referred to as *gfap:dTomato; deltaA:GFP*. This line, therefore, exhibits dTomato labeling of neural stem cells, which are radial glia cells, and GFP labeling of neural progenitors cells, in particular in the telencephalon. Anesthesia and mounting for imaging were conducted as in previous studies^26,27^. Briefly, anesthesia was induced with 0.02% MS222 (Sigma) for approximately 90 s and maintained during imaging with 0.01% MS222. Anesthetized fish were mounted in a home-made plastic dish between pieces of sponge. All experiments and husbandry were carried out in accordance with the institutional guidelines.

## Results and discussion

### Multiband SWIR source with MHz repetition rate

OPCPA is an attractive approach for developing a MHz source for 3P microscopy since it is, in principle, compatible with energetic short pulses and can enable access to long wavelengths^28,29^. Our original strategy for simultaneously generating 1.3 and 1.7 µm pulses was as follows: starting from a pump beam at *λ*_0 _= 1030 nm, we generated a signal beam at *λ*_1_ = 1700 nm and therefore a simultaneous idler beam at $\lambda _2 = \left( {\lambda _0^{ - 1} - \lambda _1^{ - 1}} \right)^{ - 1} \approx 2600$ nm, which we then frequency-doubled to obtain 1.3 µm pulses. Therefore, the main challenge was to reach µJ-range pulse energies in both beams at a MHz repetition rate. In particular, the idler amplification and subsequent frequency-doubling stages were required to produce a 1.3 µm pulse train with sufficient energy for efficient 3P imaging.

Our experimental layout is schematically illustrated in Fig. 1a and involved an YDFA pump at 1030 nm and two successive OPCPA stages (see also Materials and methods section). The pump was a commercially available YDFA delivering 50 W of average power in the form of a 1.25 MHz, 310 fs pulse train. The signal beam (1700 nm) was obtained from an SC produced by focusing one portion of the 1030 nm pump into a YAG crystal. Stretching and compression of the signal and idler pulses was achieved using Si and SiO_2_ plates. Therefore, the system relied only on compact and stable bulk elements.

One key issue in the SC generation step was to reach long wavelengths, such as 1.7 µm, before the occurrence of multi-filamentation, which has been reported to be dependent on the initial pulse duration and focusing conditions^30,31^. We obtained a good-quality SC by focusing 3 µJ, 310 fs pulses using a 150-mm lens into a 10-mm-long YAG crystal. The SC spectrum extended up to 2 µm, thereby providing a seed between 1.6 and 1.7 µm for the parametric amplification stages.

Parametric amplification is limited by beam distortions appearing at high peak and/or average pump power in a single-stage setup. We therefore implemented two stages of amplification to achieve a sufficient gain. This configuration provides additional degrees of freedom, with two MgO: PPLN crystals designed to optimize the gain and conversion efficiency. We stretched the signal pulse using a 3-mm-long Si plate before sending it to the first amplification stage. The first MgO:PPLN crystal was designed to obtain a high gain (>100 typically) with a pump pulse energy in the 1–5 µJ range. The evolution of the signal pulse energy with respect to the pump energy is shown in Supplementary Figure S1. We observed a ring-shaped distortion of the output beam profile at pump energies exceeding 3.2 µJ, corresponding to average pump powers >4 W. The threshold for the appearance of this degradation depends both on the average and peak power and may be attributed to thermal effects and nonlinear absorption^17,18^. Alternatively, linear absorption, the photorefractive effect, and pyroelectric effects can also lead to such a beam distortion at a high pump power. Taking this distortion into account, the nominal pump energy used in the first stage was 3.2 µJ, corresponding to a pump irradiance of 50 GW/cm^2^ and resulting in a gain of 130. The central signal wavelength was adjusted to be between 1700 and 1710 nm using a delay line to produce an optimal signal-idler combination for our purpose, that is, corresponding to an idler center wavelength between 2590 and 2610 nm. The signal beam bandwidth at the output of the first stage reached 107 nm full-width at half-maximum (FWHM) (Figure 1 of Supplement 1). The crystal for the second stage of amplification was chosen to have a larger aperture to sustain 33 µJ pump pulses and to provide an additional gain of 30 while avoiding beam distortion. This configuration provided a good trade-off between the amplification bandwidth and conversion efficiency. The achieved power conversion efficiencies from the pump to signal and idler were 11 and 7%, respectively. The signal was then recompressed using a 50-mm-long slab of fused silica from 220 fs down to 65 fs FWHM (Fig. 1b). The output energy at 1700 nm was 3.1 µJ, which corresponds to a peak power of approximately 4.8 GW. The stability of the output signal was typically 1.7% RMS over a few hours (Supplementary Figure S1). The beam quality of the signal (1700 nm) was well preserved, even at full pump energy. The beam profile was circular and without rings at its periphery, as seen in (Supplementary Figure S1). The quality factor *M*^2^ for the signal beam at full energy (3.1 µJ) was measured in the horizontal and vertical directions to be 1.1 and 1.15, respectively, indicating almost no degradation of the beam.

Finally, the idler was focused into a third PPLN crystal optimized for SHG (see the Materials and methods section). We obtained up to 890 mW of average power at 1300 nm in the form of 69 fs, 712 nJ pulses. The quality factor *M*^2^ for the 1.3 µm beam at full energy (0.71 µJ) was measured in the horizontal and vertical directions to be 1.7 and 1.6, respectively, indicating a small degradation of the SHG beam that can be attributed to the large conversion efficiency and parasitic conversion to 650 nm. However, even at maximum idler energy, a well-behaved beam shape (i.e., circular, uniform and without satellites) for the 1300 nm output beam was preserved (see Supplementary protocol).

Overall, the simultaneous production of two sub-70 fs pulses with a 1.25 MHz rate, one at 1.7 µm and another one at 1.3 µm, makes this laser design uniquely suited for dual-color three-photon imaging and takes advantage of the two main tissue transparency windows in the SWIR range.

### Dual-color 3P microscopy of nervous tissues

The dual SWIR source was coupled to a custom-built upright multiphoton microscope (Fig. 1c) that was also equipped with a commercial Ti:Sapphire and OPO source for comparison with two-photon microscopy (80 MHz, 130 fs, 920 or 1100 nm, see Materials and methods section).

The size and divergence for the 1.3 and 1.7 µm beams were adjusted using independent telescopes, and the beams were recombined using a dichroic mirror before entering the microscope. We estimated the axial resolutions provided by both beams by recording THG image stacks from a glass–water interface. The FWHM of the z-scans were measured to be 2.8 ± 0.1 and 3.8 ± 0.1 µm for 1.3 and 1.7 µm excitation, respectively, with an axial mismatch smaller than 0.5 µm between the two foci (Supplementary Figure S2). Under these conditions, up to 110 and 100 mW of excitation power was available after the objective for the 1.3 and 1.7 µm beams, respectively.

We first verified that our source can provide efficient deep-tissue three-photon imaging. Previous studies^3,4^ noted that the depth advantage of three-photon over two-photon excitation is maximized in densely labeled, scattering samples. We recorded two-photon and three-photon images from the cerebral cortex of fixed transgenic mice that were densely labeled with tdTomato at depths ranging from 200 to 800 µm from the tissue surface. Movie 1 and Fig. 2a confirm that the signal-to-background ratio for the 2PEF images progressively degraded with depth, whereas three-photon excitation provided an appropriate contrast to visualize cell bodies at all of the depths investigated. Figure 2a illustrates the obvious benefit provided by 3P excitation over 2P excitation at a depth of 600 µm in this sample, despite the fact that 2P imaging was carried out here using an excitation wavelength of 1100 nm, that is, under favorable conditions for deep imaging. Of note, the imaging depth in a given sample is related to the transparency of that sample, so that such a direct comparison is important to confirm the benefit of changing the excitation mode. In particular, fixed brain tissue is generally more scattering than live tissue^2^.

We then explored the performance of our SWIR source for dual-color 3P imaging. We found that combined 1.3/1.7 µm excitation enables dual-color three-photon imaging of HEK cells and mouse brain tissue labeled with several often-used GFP/RFP combinations: EGFP/mCherry, EGFP/tdTomato, and EGFP/mRFP (Fig. 2b, c).

We next imaged a fixed avian embryonic spinal cord co-labeled with EGFP and mRFP. We recorded two-color image stacks over a depth of 600 µm using two-photon and three-photon excitation sequentially (Fig. 2d, e, Movie 2–4). Two-photon imaging of EGFP and mRFP was done using 80 MHz pulse trains at 920 and 1100 nm, respectively; 3P imaging of EGFP and mRFP was done using 1.2 MHz pulse trains at 1300 and 1700 nm, respectively. In addition, the THG signal from the 1300 nm beam provided a label-free image highlighting tissue morphology. Figure 2d shows *XZ* projections of the THG, 3P-EGFP, and 2P-EGFP image stacks. As also visible in Movies 3 and 4, the two-photon contrast is rapidly degraded with depth in the central regions of the neural tube while the contrast in the three-photon and third-harmonic images remains sufficient to visualize cells and labeled processes throughout the entire stack (Movie 2). Figure 2e shows 2P and 3P-EGFP and mRFP images recorded at a depth of 500 µm and intensity profiles recorded across labeled cells. Both green and red channels exhibit superior contrast when using 3P excitation.

Finally, we explored the possibility of using our source for live three-photon imaging of dual-labeled live tissues (Fig. 3). We first recorded simultaneous GFP-RFP-THG time-lapse movies of chick embryonic spinal cord explants in samples that contained dual-compartment labeling. The tissue developed normally, revealing the dynamics of cell migration and process formation during the two-to-four hour duration of the experiments (Fig. 3b, Movies 5 and 6). We then recorded multimodal three-photon and THG images from the brain of a live adult zebrafish that contained dual-color labeling for neural stem cells and neural progenitor cells (see the Materials and methods section) from the dorsal telencephalon (pallium), which lay below the surface, underneath the skull. As shown in Fig. 3a and Movies 7 and 8, the relative distribution of the two fluorescence-labeled cell populations could be visualized at a single-cell level resolution, directly in their native environment through the skin and skull. In addition, the label-free THG signals provided a complete morphological context, highlighting skin cells, red blood cells in vessels, lipid accumulations, and skull boundaries in the intact fish head.

## Conclusions

Recent studies have revealed novel perspectives offered by SWIR three-photon excitation for deep-tissue imaging. The development of biomedical applications relies on the availability of turnkey sources that are able to provide efficient three-photon excitation of biologically relevant chromophores. Our robust OPCPA design provides simultaneous excitation in the two most promising spectral windows for deep-tissue 3P microscopy at approximately 1.3 and 1.7 µm, with near-optimal pulse characteristics. In turn, we have shown that our source can be used to readily provide simultaneous two-color 3P (and THG) images of nervous tissues that are dual-labeled with commonly used RFPs/GFPs (EGFP, mRFP, mCherry, tdTomato), with superior contrast at large depths compared to multicolor two-photon microscopy. We note that three-photon microscopy should be generally viewed as complementary to two-photon microscopy: although 3PEF microscopy can provide deeper imaging compared to 2PEF microscopy, it relies on a lower excitation repetition rate and, as a consequence, produces a smaller fluorescence flux, resulting in slower imaging or a lower signal. In addition, the use of higher-energy pulses may promote photoperturbation routes through higher-order absorption processes^32^. Systematic studies of perturbation thresholds with MHz pulse trains in live tissues are currently needed to define safe excitation windows in this regime. However, multiple applications of dual-color three-photon imaging can be envisioned in systems biology. Indeed, this approach provides a unique method to study interactions between molecular or cellular components in intact tissues at depths complementing those reached using multicolor two-photon imaging.

## Electronic supplementary material

Supplementary Information for: Dual-color deep-tissue three-photon microscopy with a multiband infrared laser

2PEF, 3PEF and THG imaging of tdTomato-labeled fixed mouse brain cortex

THG and dual-color 3PEF imaging of chick embryo spinal cord co-labeled with GFP and RFP

Comparison of 2P and 3P imaging of EGFP signals in a chick embryo spinal cord at different depths

Comparison of 2P and 3P imaging of mRFP signals in a chick embryo spinal cord at different depths

Simultaneous dual-color 3PEF and THG time-lapse imaging of developing chick embryo spinal cord tissue expressing cytoplasmic GFP labeling and nuclear RFP labeling (2 hours experiment)

Simultaneous dual-color 3PEF and THG time-lapse imaging of developing chick embryo spinal cord tissue expressing GFP and RFP labeling (4 hours experiment)

Through-skull in vivo simultaneous dual color 3PEF and THG z-stack imaging in adult zebrafish brain

3D visualization of two populations of pallial neural stem cells imaged within their native environment through skin and skull in adult zebrafish telencephalon
