# Supplementary material for: Dual-color deep-tissue three-photon microscopy with a multiband infrared laser
Source: Light Sci Appl. 2018 Jun 6;7:12. doi: 10.1038/s41377-018-0012-2 (PMC6107000; doi:10.1038/s41377-018-0012-2)
Supplement: Supplementary file 1 — Supplementary Information for: Dual-color deep-tissue three-photon microscopy with a multiband infrared laser [file 41377_2018_12_MOESM1_ESM.pdf]

Supplementary Information for:
Dual-color deep-tissue three-photon microscopy with a multiband infrared laser

Khmaies Guesmi[1,†], Lamiae Abdeladim[2,†], Samuel Tozer[3], Pierre Mahou[2], Takuma Kumamoto[3], Karolis Jurkus[1], Philippe Rigaud[1], Karine Loulier[3], Nicolas Dray[4], Patrick Georges[1], Marc Hanna[1], Jean Livet[3], Willy Supatto[2], Emmanuel Beaurepaire[2,*], Frederic Druon[1,*]

[1] Laboratory Charles Fabry, Institut d'Optique Graduate School, CNRS, Paris-Saclay University, 91128 Palaiseau, France

[2] Laboratory for Optics and Biosciences, Ecole polytechnique, CNRS, INSERM, 91128 Palaiseau, France

[3] Vision Institute, Sorbonne Universités, Université Paris 6, INSERM, CNRS, 75012 Paris, France

[4] Zebrafish Neurogenetics Unit, Developmental and Stem Cell Biology Department, Institut Pasteur, CNRS, 75015 Paris, France

† these authors contributed equally

** corresp. emmanuel.beaurepaire@polytechnique.edu, frederic.druon@institutoptique.fr*

**ITEM**	TITLE
**Protocol**	Implementation of the multiband source
**Figure S1**	Characterization of pulses energies and beams profiles
**Figure S2**	Axial resolutions of the dual color 3P microscope
**Movie 1**	2PEF, 3PEF and THG imaging of tdTomato-labelled fixed mouse brain cortex
**Movie 2**	THG and dual-color 3PEF imaging of chick embryo spinal cord co-labelled with GFP and RFP
**Movie 3**	Comparison of 2P and 3P imaging of EGFP signals in a chick embryo spinal cord at different depths
**Movie 4**	Comparison of 2P and 3P imaging of mRFP signals in a chick embryo spinal cord at different depths
**Movie 5**	Simultaneous dual-color 3PEF and THG time-lapse imaging of developing chick embryo spinal cord tissue expressing cytoplasmic GFP labelling and nuclear RFP labeling (2 hours experiment)
**Movie 6**	Simultaneous dual-color 3PEF and THG time-lapse imaging of developing chick embryo spinal cord tissue expressing GFP and RFP labeling (4 hours experiment).
**Movie 7**	Through-skull *in vivo* simultaneous dual color 3PEF and THG z-stack imaging in adult zebrafish brain
**Movie 8**	3D visualization of two populations of pallial neural stem cells imaged within their native environment through skin and skull in adult zebrafish telencephalon

Supplementary protocol – implementation of the multiband source

- Figure SP1: Multiband laser source general architecture.
- Figure SP2: Multiband laser source experimental setup.
- Experimental protocol
- Table: list of optical components

Figure SP1: Multiband laser source general architecture.

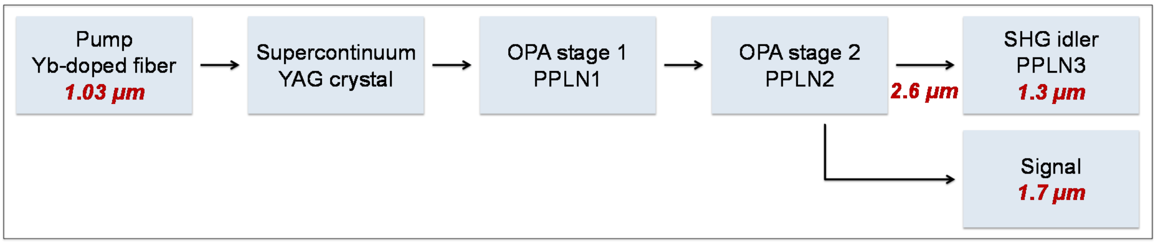

Figure SP2: Multiband laser source experimental setup.

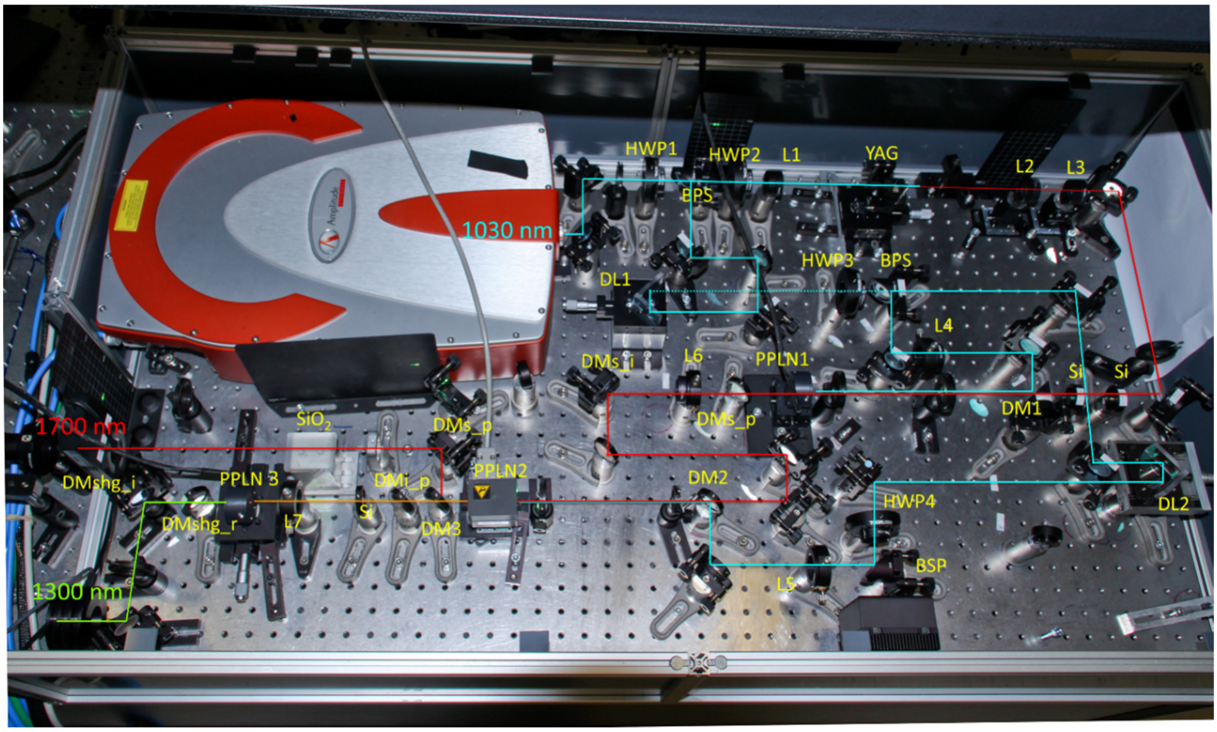

Experimental setup and beam paths. HWP: half-wave plate. BSP: beam splitter polarizer; L1=150 mm; L2=150 mm; L3=500 mm; L4=500 mm; L5=2000 mm; L6=125 mm; Si: Silicon; SiO_2: silica; DMs_p: dichroic mirror (HR 1600-1800nm, HT 1030nm); DMs_i: dichroic mirror (HR 1290-1900nm, HT 2200-4000nm), DMi_p: dichroic mirror (HR 1000-1500nm, HT 2200-3000nm); DMshg_r: dichroic mirror (HT 1300nm, HR 650nm); DMshg_i: dichroic mirror (HR 1200-1400nm, HT 2200-3000nm). DM1: dichroic mirror (HR 1030nm, HT 1600-1800 nm). DM2: dichroic mirror (HR 1030nm, and HT 1600-1800 nm). DM3: dichroic mirror (HR 1290-1900nm, HT 2200-4000nm).

Experimental protocol

Supercontinuum (SC) generation

Output from the 1030 nm commercial pump is a 2.4 mm collimated beam at 1.25MHz, with 40µJ pulse energy and 300fs pulse width.

- The 1030nm pump beam is sent through a power control module (HWP1 + PBS1). The transmitted beam is referred to as the main beam and the reflected beam as the residual pump. HWP2 is placed after the beam splitter to control the main beam polarization. Typically, HWP1 is adjusted in order to obtain ~3µJ pulse energy in the main beam.

- The main beam is then focused onto a 10-mm long YAG crystal using a 150mm lens (L1) to produce the SC. YAG crystal should be placed on a horizontally translating stage, approximately at the focal plane.

- *SC generation optimization*: a 1400nm long pass filter is placed after the YAG crystal. Using the micrometric translation stage, the YAG crystal position is adjusted in order to maximize the output power after the long-pass filter. After this adjustment, the long-pass filter is removed.

Critical step: At this stage, special care should be made to avoid multi-filamentation occurrence. This can be verified by controlling the input power (HWP1) and checking the spectrum.

OPA stage 1

This step relies on the spatial and temporal recombination of the main beam at the output of the YAG crystal and a fraction of the residual beam at 1030nm onto an MgO: PPLN (PPLN1) crystal to produce parametric amplification.

- The main beam is collimated using a 150mm lens (L2).

- The main beam is then focused onto PPLN1 with a 500mm lens (L3) in order to obtain a spot size of 190 µm × 180 µm on PPLN1.

- Prior to PPLN1, a 3 mm long Si plate is placed on the main beam path to stretch the pulse in order to increase its temporal overlap with the pump pulse.

- In parallel, a small fraction of the residual pump beam (around 3.2µJ) is focused using a 500 mm lens (L4) to obtain a spot size of 270 µm × 260 µm on PPLN1, and spatially overlapped with the main beam using a dichroic mirror (DM1, HR 1030 nm, HT 1600-1800 nm).

Critical step: The temporal and spatial overlap is a critical parameter and is adjusted by translating a roof-mirror delay line on the residual pump path (Delay line 1). The delay line setting impacts directly the output spectrum of the OPA. For that reason, a large band spectrometer (MOZZA, Fastlite, France) is used to check the output spectrum after the first OPA stage and to adjust the signal and the idler wavelength to respectively 1700±10 nm and 2600±10 nm.

OPA stage 2

This step relies on the spatial and temporal recombination of the 1700 nm signal beam (130 nJ) at the output of PPLN1 with the remaining pump from the residual pump beam (33µJ) onto an MgO: PPLN (PPLN2) crystal to produce parametric amplification.

- The pump and the idler beam after the first OPA stage are filtered out using two dichroic mirrors (DMs_p and DMs_i respectively).

- The 1700 nm signal beam is collimated with a 125mm lens (L6) in order to obtain a spot size of 1.8mm × 1.6mm on PPLN2.The residual pump beam is collimated with a L5 in order to obtain a spot size of 2 mm × 1.7 mm on PPLN2. Prior to L5, an additional HWP is placed on the residual pump beam (HWP4) to control the pump energy. Note: This beam size appears to be optimal compared to the aperture of the crystal. These "large" beam diameters allow operation at full energy without the onset of ring-like beam distortions.

- The 1700 nm signal beam and the residual pump beam are spatially overlapped using a dichroic mirror (DM2, HR 1030 nm, HT 1700 nm).

- The temporal overlap in the second OPA stage is set using a second delay line (DL2) on the residual pump beam.

Idler frequency doubling

Second harmonic generation (SHG) of the idler beam at 2600 nm is performed on a third PPLN crystal (PPLN3) to reach the wavelength of interest (1300 nm).

- The 2600 nm idler is separated from the 1700nm signal beam with a dichroic mirror (DM3)

-The idler is recompressed using a 5mm-long Si plate providing positive dispersion.

- After filtering out the residual pump from the idler beam (Dmi_p), the idler is focused onto PPLN3 with a 100 mm CaF_2 lens (L7).

Note 1: Since the 2600 nm idler beam can be absorbed by air humidity, we minimized the propagation distance between the OPA output and the frequency doubling stage.

Note 2: We have tested other nonlinear crystals for SHG such as AGS, but we found that in our conditions PPLN is the best in terms of efficiency and beam quality.

Abbreviations

L: lens. BPS: beam splitter polarizer. HWP: half-wave plate. SC: supercontinuum. YAG: yttrium aluminium garnet. OPA: optical parametric amplification. DL: delay line. SHG: second harmonic generation. AGS: AgGaS2.

**Table – list of optical components**

HWP1/2/3	Zero order half wave plates, AR coating: 1030 nm **Thorlabs, USA.**
BSP	Beam splitter polarizer, Wavelength design: 1030 nm, **Layertec, Germany.**
L1	Plano convex lens, f=150 mm, B coating 650-1050 nm, **Thorlabs, USA.**
L2	Plano convex lens, f=150 mm, A coating 1050-1700 nm, **Thorlabs, USA.**
L3	Plano convex lens, f=500 mm, A coating 1050-1700 nm, **Thorlabs, USA.**
YAG	YAG (Yttrium Aluminium Grenat) crystal, BBAR/BBAR 1030-1800 nm, **Eksma Optics, Lithuania.**
Delay Line (DL1/2)	HR right angle retroreflector, design wavelength: 1020-1090 nm, AOI: 45°, Reflection >99 %, GDD<20 fs^2, **Altechna, Lithuania.**
L4	Plano convex lens, f=500 mm, B coating 650-1050 nm, **Thorlabs.**
Si	Silicon window, AR/AR 1300-4200 nm, (AOI=0 °) **Altechna, Lithuania.**
DM1	Dichroic mirror, HR 1030 nm, and HT 1600-1800 nm, **Altechna, Lithuania.**
PPLN1	MgO:PPLN crystal, Coating:1030-1700 nm (R<2 %) on S1, and AR coating at 1030-1060 (R<0.5 %) / 1400-1800 (R<1 %) / 2200-3000 (R<5 %) nm on S2, QPM periods (µm): 29.52 / 29.98 / 30.49 / 31.02 / 31.59, **Covesion Ltd, UK.**
L5	Plano convex lens, f=2000 mm, B coating 650-1050 nm, **Eksma Optics**, **Lithuania.**
L6	Plano convex lens, f=125 mm, A coating 650-1050 nm, **Thorlabs, USA.**
DMs_i	Dichroic mirror, HR1290-1900 nm, HT2200-4000 nm, **Laser Optik, Germany.**
DM2	Dichroic mirror, HR1030 nm, and HT 1600-1800 nm, **Altechna, Lithuania.**
PPLN2	MgO:PPLN crystal, Material 5 mol%, QPM period(µm): 29.98 / 30.49 / 31.03, Coating:1030-1700 nm(R<2 %) on S1, and AR coating at 1030-1060 (R<0.5 %) / 1400-1800 (R<1 %) / 2200-3000 (R<5 %) nm on S2, **HCPhotonics.**
DM3	Dichroic mirror, HR1290-1900 nm, HT2200-4000 nm, **Laser Optik, Germany.**
DMs_p	Dichroic mirror, HR@1030 nm, HT@1600_1800 nm, **Altechna, Lithuania.**
DMi_p	Dichroic mirror, HR1000-1500 nm, HT2200-3000 nm, **Laser Optik, Germany.**
L7	Caf2 Focal lens, f=100 mm, D coating 1650-3000 nm **Eksma Optics, Lithuania.**
SiO_2	Fused silica window, AR/AR1600-1800 nm, AOI=0°, **Altechna, Lithuania.**
PPLN3	PPLN crystal for SHG (item: MSHG2600), length: 1mm, periods: 34.00 / 34.80 / 35.50 / 35.80 / 35.97 µm, **Covesion Ltd, UK.**
DMsgh_r	Dichroic mirror, HT1300 nm, HR650 nm, **Thorlabs, USA.**
DMsgh_i	Dichroic mirror, HR1200-1400 nm, HT2200-3000 nm, **Altechna, Lithuania.**

Figure S1. Characterization of pulses energies and beams profiles.

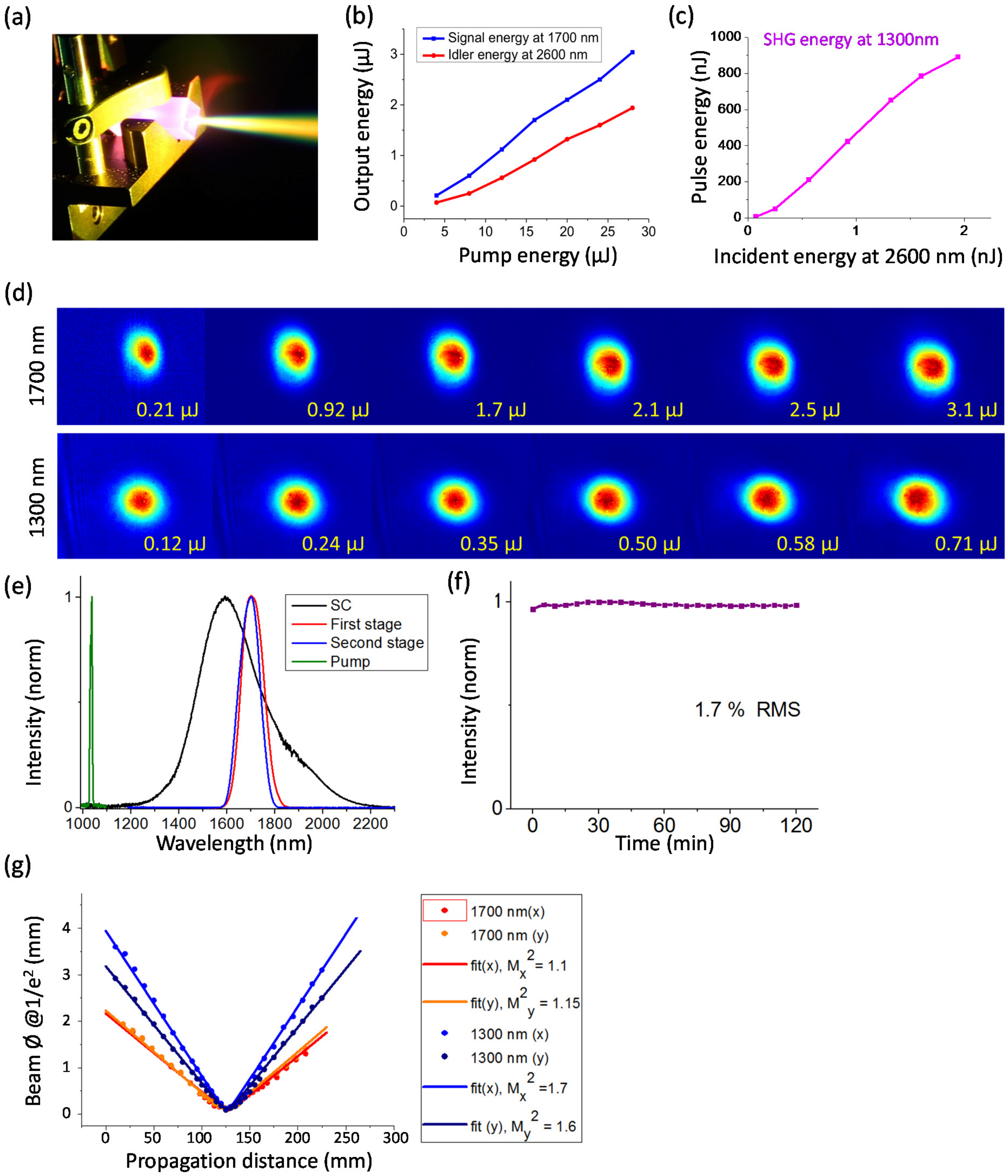

Figure S1. Characterization of pulses energies and beams profiles. (a) Supercontinuum generation in the bulk YAG crystal. (b) Evolution of output energy at 1700 nm and 2600 nm as a function of pump energy in the second OPA stage. (c) Evolution of SHG output energy at 1300 nm as a function of incident energy at 2600 nm. (d) Measured beam profiles with increasing output pulse energy. (e) Optical spectra of pump (green), supercontinuum after Si stretcher (black), output of OPA stage 1 (red), output of OPA stage 2 (blue). (f) Temporal stability of the 1700 nm beam power. (g) Quality factor M^2 measurement of the two beams at 1300 nm and 1700 nm.

Figure S2. Axial resolutions of the dual color 3P microscope.

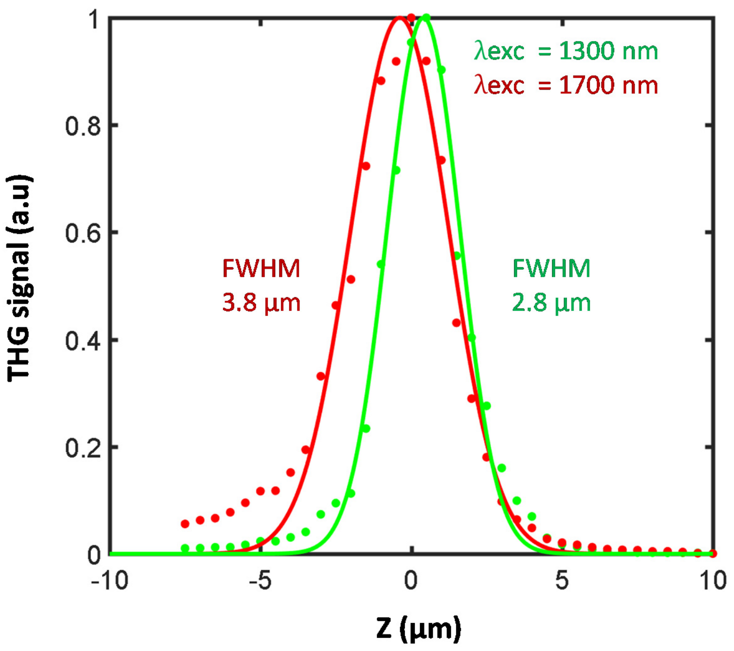

Figure S2. Axial resolution during two-color 3PEF imaging. Axial resolutions and axial foci mismatch estimated from THG z-stacks through a water-glass interface with the 1300 nm beam (green) and the 1700 nm beam (red).

Movies

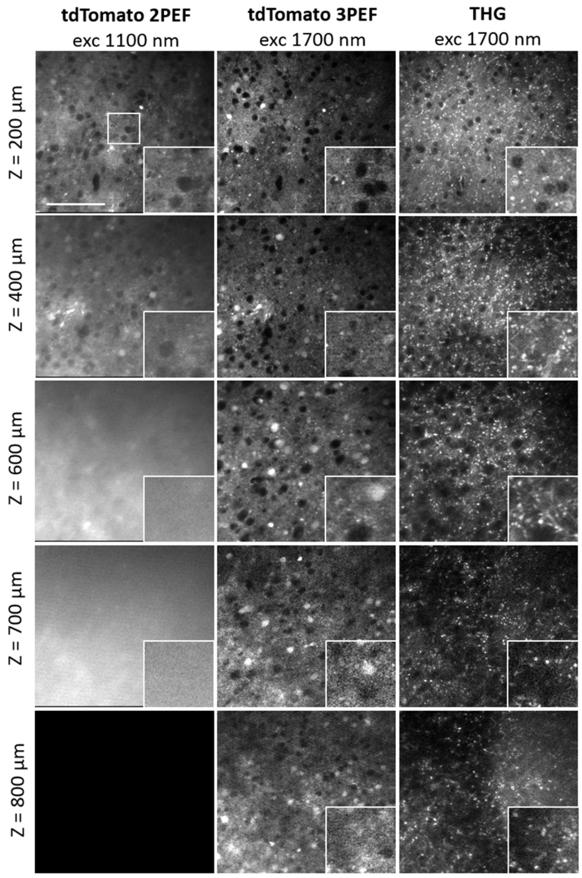

Movie 1. 2PEF, 3PEF and THG imaging of tdTomato-labelled fixed mouse brain cortex. Image stacks were recorded sequentially with 1100 nm excitation (2PEF) and with 1700 nm excitation (3PEF and THG). Images are intensity-normalized in each plane for contrast comparison. Conditions for 2P imaging: 1100 nm, 80 MHz, 40 to 150 mW on the sample and 1 to 16 accumulations depending on imaging depth. Conditions for 3P imaging: 1700 nm, 1.2 MHz, 2 to 100 mW at the sample surface depending on imaging depth, 1 accumulations. Time per pixel, 10 µs. Scale bar, 100 µm.

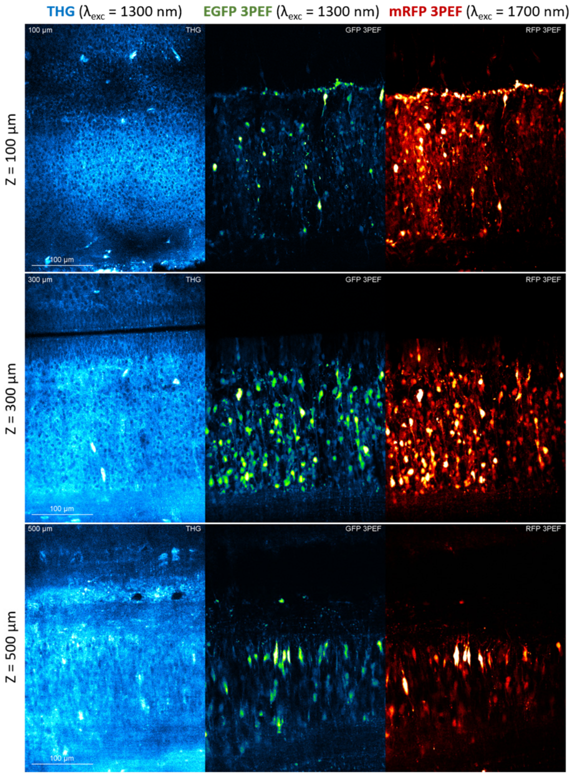

Movie 2. THG and dual-color 3PEF imaging of chick embryo spinal cord co-labelled with GFP and RFP. Image stacks were recorded with 1300 nm (EGFP and THG) and 1700 nm (mRFP) excitation. Images are intensity-normalized in each plane for contrast comparison. Conditions for 3P imaging: 1300 or 1700 nm, 1.2 MHz, 15 to 100 mW at the sample surface depending on imaging depth. Time per pixel, 5 µs. Scale bar, 100 µm.

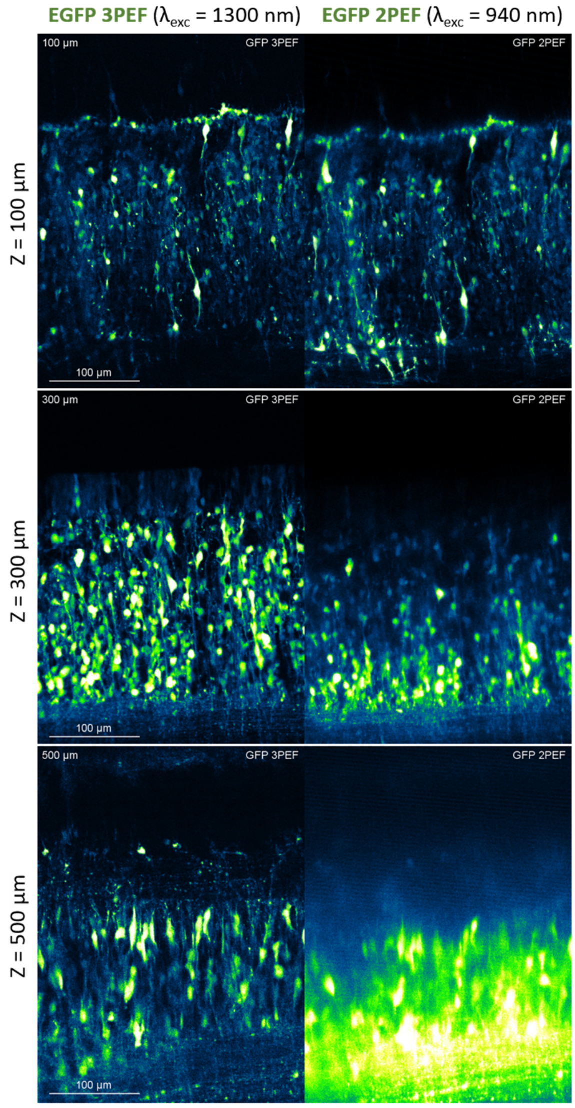

Movie 3. Comparison of 2P and 3P imaging of EGFP signals in a chick embryo spinal cord at different depths. The transgene electroporation strategy (see methods) results in a dense labeling at depths between 200 and 500 µm of the 650 µm-diameter neural tube. Conditions for 2P imaging: 940 nm, 80 MHz, 10 to 50 mW at the sample surface and 1 to 4 accumulations depending on imaging depth. Conditions for 3P imaging: 1300 nm, 1.2 MHz, 15 to 100 mW at the sample surface. Time per pixel, 5 µs. Scale bar, 100 µm. Images are intensity-normalized in each plane for contrast comparison.

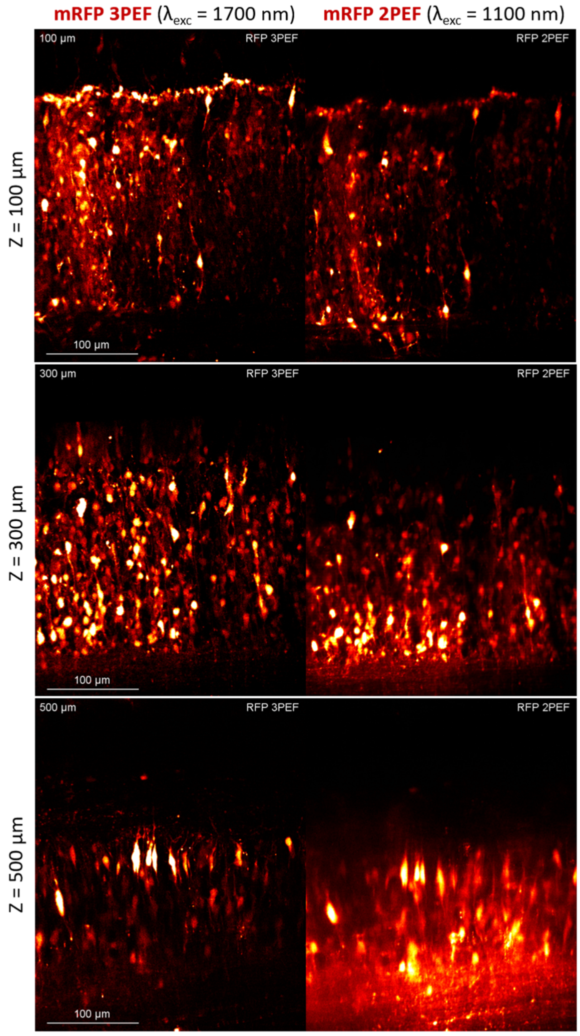

Movie 4. Comparison of 2P and 3P imaging of mRFP signals in a chick embryo spinal cord at different depths. The transgene electroporation strategy (see methods) results in a dense labeling at depths between 200 and 500 µm of the 650µm-diameter neural tube. Conditions for 2P imaging: 1100nm, 80 MHz, 10 to 150 mW at the sample surface depending on imaging depth. Conditions for 3P imaging: 1700nm, 1.2 MHz, 15 to 100 mW at the sample surface and 1 to 4 accumulations depending on imaging depth. Time per pixel, 5 µs. Scale bar, 100 µm. Images are intensity-normalized in each plane for contrast comparison.

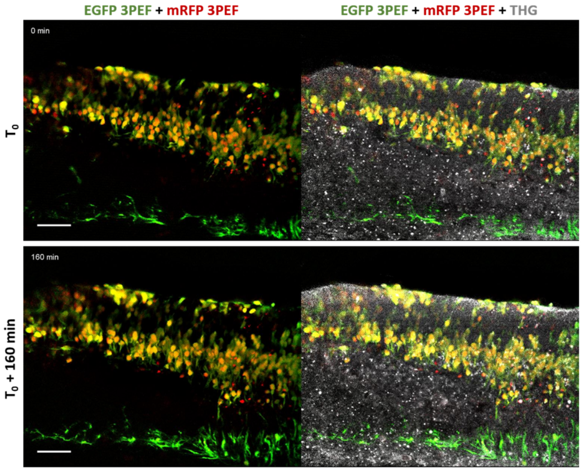

Movie 5. Simultaneous dual-color 3PEF and THG time-lapse imaging of developing chick embryo spinal cord tissue expressing cytoplasmic GFP labelling and nuclear RFP labeling (2 hours experiment). A 2-hours movie was recorded with one image every 10 minutes. Power after the objective was approximately 30 mW for each beam. Pixel size, 0.55µm. Time per pixel, 5 µs. Scale bars, 50 µm. Normal developmental was observed during the experiment.

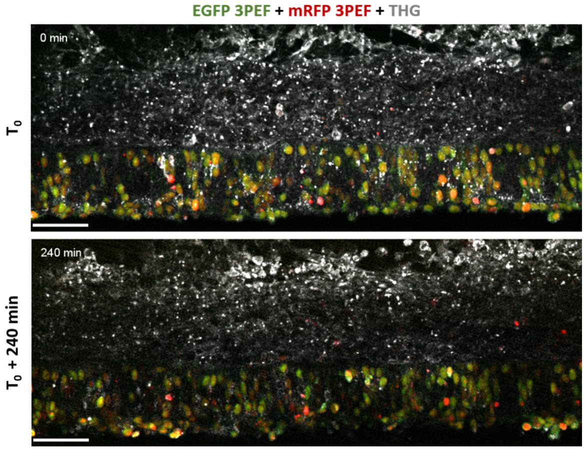

Movie 6. Simultaneous dual-color 3PEF and THG time-lapse imaging of developing chick embryo spinal cord tissue expressing GFP and RFP labeling (4 hours experiment). A 4-hours movie was recorded with one image every 15 minutes. Power at the sample surface was approximately 25 mW and 15mW for the red and green beams respectively. Pixel size, 0.54 µm. Time per pixel, 5 µs. Scale bars, 50 µm. Normal developmental was observed during the experiment.

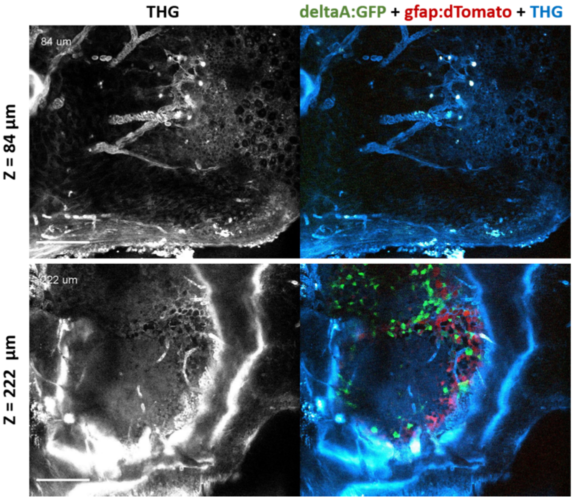

Movie 7. Through-skull *in vivo* simultaneous dual color 3PEF and THG z-stack imaging in adult zebrafish brain. Images acquired ever 3 µm. Power ranged from 20mW to 50mW for each beam, depending on the imaging depth. Pixel size, 0.54 µm. Time per pixel, 5 µs Scale bars: 100 µm.

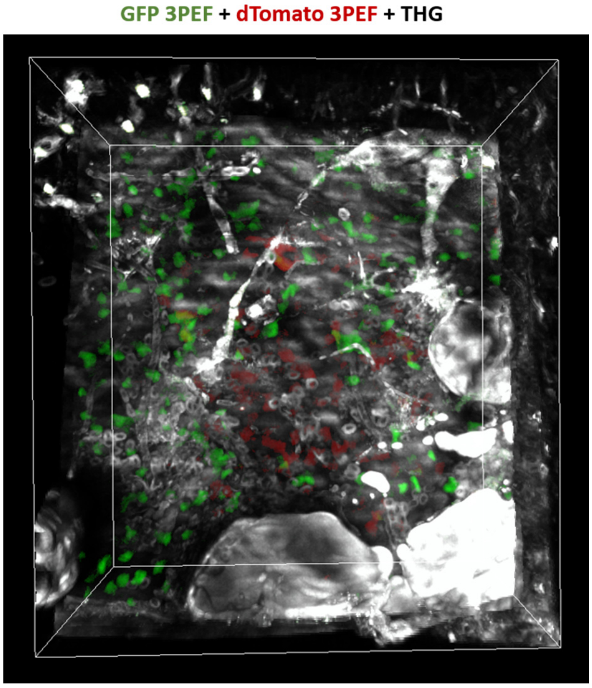

Movie 8. 3D visualization of two populations of pallial neural stem cells imaged within their native environment through skin and skull in adult zebrafish telencephalon. Simultaneous red/green 3PEF and THG imaging. Images acquired ever 3 µm. Power ranged from 20mW to 50mW for each beam, depending on the imaging depth. Pixel size, 0.54 µm. Time per pixel, 5 µs.
